# Supplementary material for: A wireless W-band 3D-printed temperature sensor based on a three-dimensional photonic crystal operating beyond 1000 ∘C
Source: Commun Eng. 2024 Sep 23;3:137. doi: 10.1038/s44172-024-00282-5 (PMC11420215; doi:10.1038/s44172-024-00282-5)
Supplement: Supplementary file 2 — Supplementary Materials [file 44172_2024_282_MOESM2_ESM.pdf]

Supplementary information: A wireless W-band 3D-printed temperature sensor operating beyond 1000 °C

Contents

1 Supplementary Note S1: Clutter suppression via high-Q cavities 2

2 Supplementary Note S2: 3D photonic crystal dimensions 3

3 Supplementary Note S3: Setup for temperature measurements 5

4 Supplementary Discussion S4: Range readout estimation 6

5 Supplementary Discussion S5: Estimation of τ_t 7

Supplementary Note S1: Clutter suppression via high-Q cavities

High-Q cavities feature a long ringing response in the time domain, that can be taken advantage of to retrieve their resonance frequency. This is particularly useful in highly cluttered environments, where the presence of clutter might mask the response of the sensor.

Consider Supplementary Figure 1, where a sketch of a dynamic scenario is depicted for a sensor implementing a single cavity, for simplicity. After the cavity is excited by an interrogation signal, it starts to slowly re-radiate its resonance response towards the interrogator, which might be mixed with clutter (gray peaks). In the case of an indoor situation, this might be people, walls, obstacles etc. Without time gating, the clutter and sensor's structural mode add to the cavities' reradiated power, resulting in a dip or notch in the received frequency-domain spectrum, or even its complete masking.

However, it is possible to profit from the fact that environment echoes are short-lived, while the cavity backscatters its response over a long time, outlasting them. To achieve so, it is enough to isolate the ringing tail of the cavity in time-domain, as shown by the green rectangle. In this case, the cavity can be considered as the only source in the environment, which radiates at its resonance frequency. This is perceived as a peak in the frequency domain, which corresponds to the sensor's response. Furthermore, clutter has been filtered out by this technique.

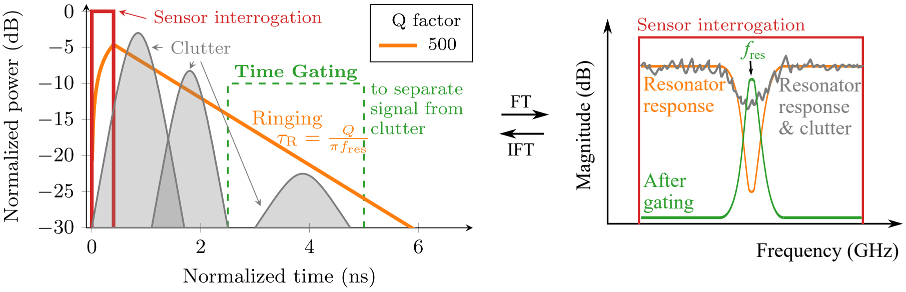

Supplementary Figure 1: Sketch depicting the operating principle of clutter suppression based in high-Q cavities.

Supplementary Note S2: 3D photonic crystal dimensions

The dimensions for the 3D photonic crystal presented in this work are summarized in Supplementary Figure 2, whereas the design values for the cavities are presented in Supplementary Figure 3 and Supplementary Table 1

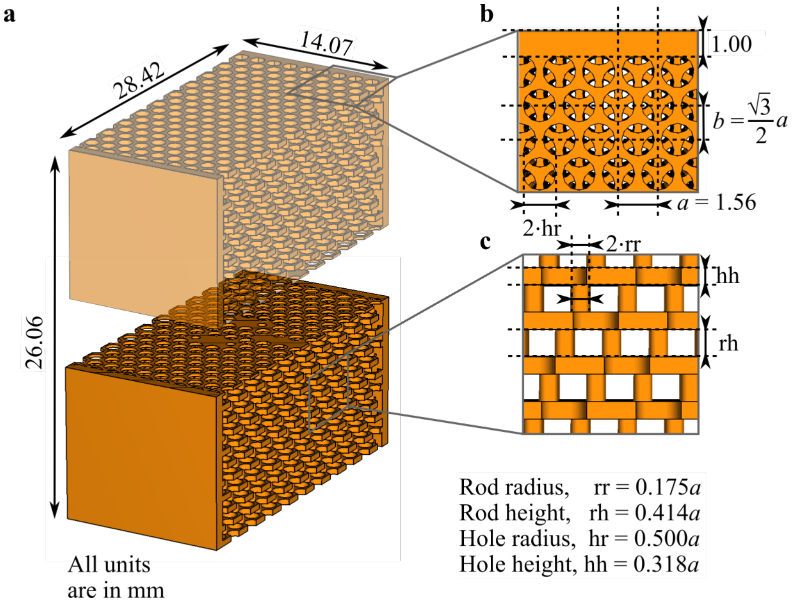

Supplementary Figure 2: Dimensions of the 3D photonic crystal structure. **(a)** Overall dimensions. **(b)** Upper view, with the lattice constant a marked. **(c)** Side view of the photonic crystal.

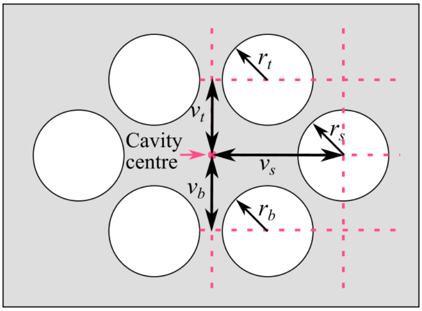

Supplementary Figure 3: Sketch of an exemplary cavity with marked design parameters.

Supplementary Table 1: Design parameters for the two cavities embedded within the 3D PhC

Parameter	Cav. 1	Cav. 2	Description
f_{res} (GHz)	83.85	85.50	Resonance frequency
v_{s} (mm)	1.66	1.46	Displacement for side holes
r_{s} (mm)	0.40	0.40	Radius for side holes
v_{t} (mm)	1.76	1.76	Displacement for top holes
r_{t} (mm)	0.59	0.59	Radius for top holes
v_{b} (mm)	1.76	1.76	Displacement for bottom holes
r_{b} (mm)	0.59	0.59	Radius for bottom holes

Supplementary Note S3: Setup for temperature measurements

The measurement setup employed for the temperature measurements is presented in Supplementary Figure 4. The sensor - formed by the 3D photonic crystal with the flattened lens on top - was placed inside the furnace. A fan was added for cooling, as it was found that the springs holding the furnace's door closed softened up after approximately 1000 °C. This implied that the door opened slightly, which could potentially heat up the measuring antenna. The effectivity of the fan on keeping the measurement equipment at room temperature was confirmed via multiple measurements with a thermal camera.

For automatic measurements, voltage out of the furnace's thermopar was measured with a voltmeter, and the temperature was estimated by reading out the corresponding voltage and comparing it to calibration curves of voltage versus temperature previously established. MATLAB was used to automatize the process.

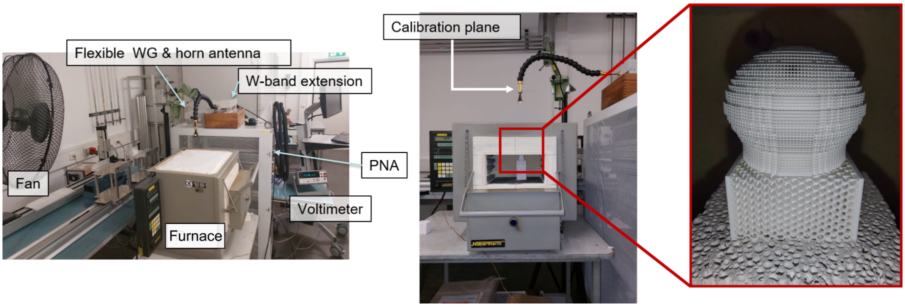

Supplementary Figure 4: Setup for the temperature measurements. (a) Picture showing the whole measurement setup from far away. **(b)** Close-up showing the sensor inside the furnace and marking the calibration plane that was employed, to remove the effect of the flexible W-band waveguide. **(c)** Close-up to the sensor itself, showing that the lens is placed on top, and centred, on the photonic crystal.

Supplementary Discussion S4: Range readout estimation

As presented in the main body of the publication, the readout range of the sensor with our measurement setup is limited to 1 m at room temperature and to approximately 0.5 m at 1200 °C, which is a short range for indoor applications. However, its measured radar cross-section is of −20 dB m^2, which can be employed to predict the readout range when changing system parameters such as the reader's antenna gain, sensitivity, transmitted power, etc.

The maximum readout distance for the sensor can be estimated by employing the radar range equation (Eq. 1).

$$R_{\mathrm{max}} = \sqrt[4]{\frac{\Delta P}{\gamma_{\mathrm{min}}} \cdot \frac{G^2 \lambda^2 \sigma}{(4\pi)^3}} \tag{1}$$

On this equation, ΔP stands for the difference between the transmitted power and the received power without a sensor, $\Delta P = P_{\mathrm{t}}/P_{\mathrm{rx,\ no\ sensor}}$. The minimum value for $P_{\mathrm{rx,\ no\ sensor}}$ is determined by the noise floor of the reader. I.e., ΔP describes the available dynamic range of the measurement equipment. Then, γ_{min} accounts for a minimum required contrast between the backscattered power by the sensor and the noise floor, so the sensor can be accurately detected, $\gamma_{\mathrm{min}} = P_{\mathrm{rx,\ sensor}}/P_{\mathrm{rx,\ no\ sensor}}$. Finally, G describes the transmitter/receiver antenna gain, λ corresponds to the operating wavelength and σ is the radar cross-section of the sensor.

One simple approach to extend the readout range, according to Eq. 1, involves raising G, where each 6 dB increase doubles the preceding readout range. The horn antenna employed in our measurement setup has a G of 23 dBi, whereas the W-band lens antenna in [1] has 35 dBi. Furthermore, commercially available Cassegrain reflector antennas reach values of 50 dBi and higher. Supplementary Figure 5 presents the maximum readout distance of the sensor against the coefficient $\Delta P/\gamma_{\mathrm{min}}$, representing the results for the aforementioned antennas. This maximum range is presented for the temperature range between room temperature (T = 21 °C) and the maximum measured temperature (T = 1200 °C), as the increased dielectric losses with temperature decrease the sensor's backscattered power by approximately 7 dB.

For our measurement setup, ΔP = 70 dB, while γ_{min} is set to 10 dB to guarantee a large enough contrast between the sensor's frequency peaks and the noise floor. It is noticeable the short R_{max} when the horn antenna in our laboratory is employed (blue lines), of 0.75 m for T = 1200 °C. However, this increases rapidly when the considered antenna is the aforementioned lens antenna or Cassegrain reflector antenna, to 3.5 m and 18 m, respectively. It should be mentioned that the maximum readout distance can also be increased by decreasing the noise floor. For example, the clutter suppression method detailed in [2] decreases the noise floor by 10 dB compared to time-gating, and thus increases the product $\Delta P/\gamma_{\mathrm{min}}$ by 10 dB (dashed gray line in Fig. 5).

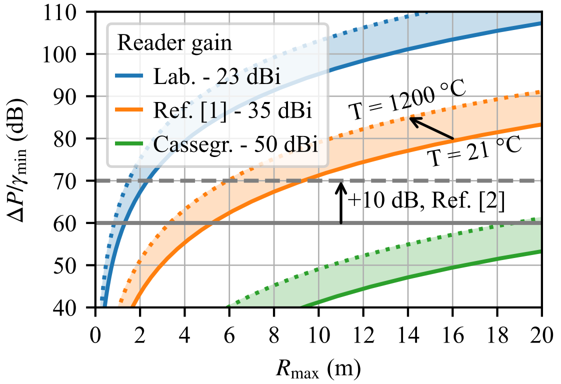

Supplementary Figure 5: Maximum achievable range

Supplementary Discussion S5: Estimation of τ_t

An estimation of the time required for the sensor to react to temperature changes is given in this section, assuming that the sensor is in contact with solid metallic surfaces on its sides. Further, it is assumed that this metal and Alumina have perfect thermal conductivity and that there is no heat radiation in the environment, so that the heat transfer occurs only at the metal-ceramic interface.

When the environment increases its temperature ΔT, the sensor temperature exponentially increases over time according to [3]:

$$T_\mathrm{sensor}(t) = T_0 + \Delta T \cdot (1 - e^{-t/\tau_\mathrm{t}}), \text{ with } \tau_\mathrm{t} = \frac{\rho \cdot c_\mathrm{p} \cdot V}{h_\mathrm{c} \cdot A_\mathrm{s}} \tag{2}$$

With T_0 the initial temperature and τ_t the time constant of the sensor. Further, ρ corresponds to the density of the ceramic, c_p to the specific heat, V the sensor's volume, h_c the interfacial heat transfer conductance at the metal-ceramic interface and A_s the contact surface area between the metallic parts and the sensor.

Assuming 99 % Al_2O_3, the values of the previous parameters are $\rho = 3890\,\mathrm{kg\,cm^{-3}}$, $c_\mathrm{p} = 880\,\mathrm{J\,kg^{-1}\,K^{-1}}$ and h_c varies between $1500\,\mathrm{W\,m^{-2}\,K^{-1}}$ to $8500\,\mathrm{W\,m^{-2}\,K^{-1}}$, depending on the surface finishes and pressure between the metal and ceramic [3].

In order to estimate τ_t, some assumptions are made. First, the lens is excluded from these calculations, as the shift in f_res regarding temperature occurs when the cavities (and hence the PhC structure) are heated up. Second, due to its large porosity, the PhC has a filling factor, η_fill of 11.26 %. Hence, V is calculated as $V = \eta_\mathrm{fill} \cdot V_\mathrm{block}$, where V_block corresponds to the volume of the PhC if it was completely solid. Do note that this simplification assumes that there are no input waveguide or cavities implemented within the PhC

structure. Third, it is assumed that several sides of the PhC are enclosed by a thin Alumina slab, which in turn is in contact with a metallic plate. This is a feasible situation in reality, as the three-dimensional electromagnetic bandgap isolates the cavities from external influences, as mentioned in the main publication.

With these assumptions, four different cases are used to calculate τ_t, following the sketch in Supplementary Figure 6.

1. Only the bottom side of the PhC is in contact with metal.
2. The bottom side and two of the opposite sides of the PhC act as heating sources.
3. All sides (but for the one on which the lens is placed) are in contact with metal.

As τ_t decreases for a larger A_s, case 3 will be the one to present a smaller τ_t. The results are summarized in Table 2. Following Eq. 2, for $t = \tau_t$, the difference between the temperature of the PhC and contact metal pads has been decreased by 63 %. It can be assumed that the PhC has heated up to the target temperature after $3\tau_t$ (95 %). Considering the best-case scenario (case 3), this implies that the PhC takes between 2 min to 11.8 min to heat up completely, depending on h_c.

The formula above assumes that there is no convection due to heated air. However, the large porosity of the PhC implies that in real use-cases, such as during a fire or inside a turbine, the superheated air seeps in the PhC structure, allowing for a faster τ_t. As a preliminary measurement, a heat gun spewing air at its medium air flow strength and 650 °C has been placed at a distance of 15 cm from the sensor. The final temperature of the sensor, measured with a thermal camera, was 125 °C. The sensor was then rapidly cooled down by switching the heat gun to operate at 50 °C, with the sensor reaching a temperature of 51 °C after 2.2 min. These results give an estimated τ_t of

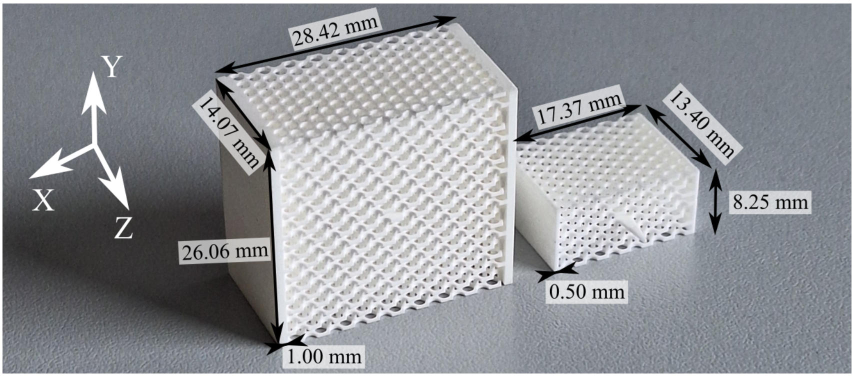

Supplementary Figure 6: Side by side comparison of the PhC employed in this work and a smaller one that still preserves the cavities' responses.

approximately 30 s. However, do note that, to accurately measure τ_t, the sensor should be placed directly in a pre-heated environment, which is currently not feasible due to the need for alignment between the PhC structure and the lens antenna.

As an observation, it is possible to decrease τ_t by minimizing the coefficient V/A_s, while keeping the advantages of a three-dimensional bandgap, by substituting the lens by a different antenna, such as a rod antenna. In this case, the overall volume of the PhC can be minimized, as there is no need to provide for a flat support for the lens. An example of such structure is presented in Supplementary Figure 6, where it can be appreciated that the PhC is significantly smaller. In this case, the best-case scenario following Supplementary Table 2 decreases the total heating time to 1 min, at the cost of sacrificing readout range, due to employing an antenna with a smaller aperture. Nevertheless, this showcases the different situations where this tag could be potentially used, either as a cooperative indoor radar target, or even as a temperature sensor, able to continuously operate at extremely high temperatures.

Supplementary References

[1] Pohl, N. *et al.* Radar measurements with micrometer accuracy and nanometer stability using an ultra-wideband 80 GHz radar system. *2013 IEEE Topical Conference on Wireless Sensors and Sensor Networks (WiSNet)* 31–33 (2013). https://doi.org/10.1109/WiSNet.2013.6488624 .

[2] Sánchez-Pastor, J. *et al.* Clutter Suppression for Indoor Self-Localization Systems by Iteratively Reweighted Low-Rank Plus Sparse Recovery. *Sensors* **21** (20) (2021). https://doi.org/10.3390/s21206842 .

[3] Lienhard, J. H., IV & Lienhard, J. H., V. *A Heat Transfer Textbook* 5th edn (Dover Publications, Mineola, NY, 2019). URL http://ahtt.mit.edu.

Supplementary Table 2: Calculated values for τ_t in the different scenarios considered

		**Case 1**	**Case 2**		**Case 3**
			Sides in X dir.	Sides in Y dir.	
**PhC**	$h_{c,min}$	722 s	363 s	347 s	235 s
	$h_{c,max}$	121 s	64 s	61 s	41 s
**Small PhC**	$h_{c,min}$	655 s	257 s	154 s	113 s
	$h_{c,max}$	116 s	45 s	27 s	20 s
